# Axial Compressive Properties of Self-Compacting Concrete Filled Steel Tube Short Columns with Ground Desulfurization Slag

**DOI:** 10.3390/ma15186306

**Published:** 2022-09-11

**Authors:** Lan Liu, Yuhao Liu, Zhi Cheng, Xinrong Cheng, Hongping Zhang

**Affiliations:** School of Science, North University of China, Taiyuan 030051, China

**Keywords:** desulfurization slag, axial compressive property, self-compacting concrete filled steel tube, load–deformation relationship, ultimate bearing capacity

## Abstract

Desulfurization slag (DS) is the solid waste discharged from the bottom of the circulating fluidized bed (CFB) boiler. It has good pozzolanic activity, self-hardening property and large expansibility. In this paper, ground desulfurization slag (GDS) is used as mineral admixture to replace cement to prepare self-compacting concrete (SCC). In order to find out the influence laws of different factors on the axial compressive properties of the self-compacting concrete filled steel tube (CFST), seven types of SCC are prepared and nine groups of CFST short column are fabricated. Filling ability test, compressive strength test and axial compressive test are performed. The filling ability and the compressive strength of the SCCs are investigated, and the axial compressive properties of the CFSTs are researched. The results show that the amount of polycarboxylate superplasticizer (PS) increases with the amount of GDS, and the addition of GDS decreases the 3d, 7d and 28d compressive strength of the SCCs. The optimum amount of GDS for SCCs and CFSTs is 30%. When the amount of GDS is 30%, the ultimate bearing capacity of CFST short column (GP3) is the highest, which is 33.6% higher than that of GP1 without GDS. The influence law of the GDS’s amount on the CFSTs’ ultimate bearing capacity is quite different from that of the GDS’s amount on the SCCs’ compressive strength. The ultimate bearing capacity of CFSTs can be significantly improved by adding GDS. Sodium sulfate can improve both the compressive strength of the SCC and the bearing capacity of the CFST.

## 1. Introduction

Self-compacting concrete (SCC) refers to the concrete with great fluidity, good uniformity and stability, which can flow by virtue of its own weight and fill the whole formwork space without vibration during pouring. Compared with ordinary concrete, fresh SCC has the properties of good filling, clearance passing and segregation resistance [1]. After hardening, the SCC has good resistance to external environment erosion [2,3]. Mineral admixtures are usually used to improve the properties of SCC, such as fly ash, granulated blast furnace slag and so on [4,5,6]. Due to the large amount of cement used, SCC has larger shrinkage than ordinary concrete. In order to compensate the shrinkage of SCC, expansion agents are added by researchers [7,8,9].

Concrete filled steel tube (CFST) refers to the composite component formed by pouring concrete into the steel tube. It is a new type of composite structure developed by combining the advantages of steel tube and concrete [10]. Pouring SCC into steel tube can not only guarantee the compactness of the core concrete and simplify vibration process of the core concrete but also reduce the construction intensity, cost and urban noise pollution. In recent years, high performance concrete filled steel tube (HCFST) has been widely used in high-rise buildings, long-span bridges, industrial buildings, transmission towers and underground structures and has achieved good economic and social benefits [11]. In the preparation of HCFST, due to the high fineness of high grade cement, silica fume, fly ash and other mineral admixtures contained in HCFST, large volume shrinkage will occur in the hydration and hardening process. The shrinkage of core concrete has a significant influence on bonds between the steel tube and the core concrete [12]. In HCFST columns, ultra-high-performance concrete (UHPC) has a large shrinkage and could easily be deboned from the steel tube, resulting in a failure of the effective lateral restraint of the steel tube to the core concrete [13]. Using an expansive agent could enhance the elastic modulus, yield and ultimate strengths of the CFST columns [14]. The self-prestress, due to expansion, can not only compact the microstructure of core UHPC but also enhance the bonding between UHPC and the steel tube [15]. A calcium sulfoaluminate expansion agent, calcium oxide expansion agent and compound expansion agent are commonly used as expansion agents in construction engineering. Magnesium oxide is also used as expansion agent in recent research work [16]. The price of expansion agent is more expensive than cement. The addition of an expansion agent increases the costs of CFST.

Circulating fluidized bed (CFB) combustion technology is a new generation of efficient and low pollution combustion technology, which developed rapidly in the world in recent years [17]. However, the in-furnace desulphurization technology of CFB boilers requires the addition of a desulphurizer (usually limestone), which produces a large amount of desulphurization ashes, including ash and slag. The desulphurization ash (DA) is the residue collected from the flue, and the desulphurization slag (DS) is the residue discharged from the bottom of the furnace [18]. Studies have shown that the amount of the desulfurization ashes from CFB boiler is about 30%~40% more than fly ash from pulverized coal furnace [19]. However, the utilization rate of the desulphurization ashes is less than 10%, and most of them are in the state of stockpiling [20].

Studies showed that desulfurization ashes have good pozzolanic activity, self-hardening property and large expansibility [21]. Desulfurization ashes contain much SiO_2_ and Al_2_O_3_, which forms the foundation as supplementary cementitious material. They can be used to replace cement for preparing green, low-carbon building materials, which has low energy consumption in cement production and low CO_2_ emissions [22]. However, the f-CaO and SO_3_ in desulfurization ashes can lead to expansion even crack, which cause difficulties for large-scale resource utilization. Desulfurization ashes should be modified before use to eliminate the destruction caused by expansion. Physical grinding can not only raise the desulfurization ashes activity, but also decrease the expansive destruction. By grinding, the dense structure of Ⅱ-CaSO_4_-wrapped CaO is broken, and CaO is released [23,24]. In alkaline environment, Ⅱ-CaSO_4_ can dissolve quickly to form ettringite, which enables the expansion to be released at an early stage [25]. As a chemical activator, sodium sulfate can promote the dissolution speed of Ⅱ-CaSO_4_ and improve the early strength of the cementitious system [26]. Research shows that moderate desulfurization ashes have not done harm to cements or concretes, in fact, they have even improved their strength and compensated their shrinkage [27,28].

The use of circulating fluidized bed desulfurization slag (CFBDS) to replace cement can not only reduce CO_2_ emissions but also solve many environmental problems caused by the large amount of CFBDS stockpiles [29]. The use of CFBDS in CFST columns can not only reduce the costs but also compensate the core concrete’s shrinkage and improve CFST’s mechanical properties. In this paper, seven types of SCCs were prepared by using the desulfurization slag as a mineral admixture to replace cement. The filling ability and the compressive strength of the SCCs were investigated. Nine groups of CFST short column were fabricated and the axial compressive properties of the CFSTs were researched. The influence laws of different influence factors on the axial load-deformation and the ultimate bearing capacity of CFSTs were revealed. The research results can provide a new direction for the utilization of desulfurization ashes and provide a new method for the preparation of a concrete-filled steel tube.

## 2. Materials and Methods

### 2.1. Raw Materials

**Figure 1:**
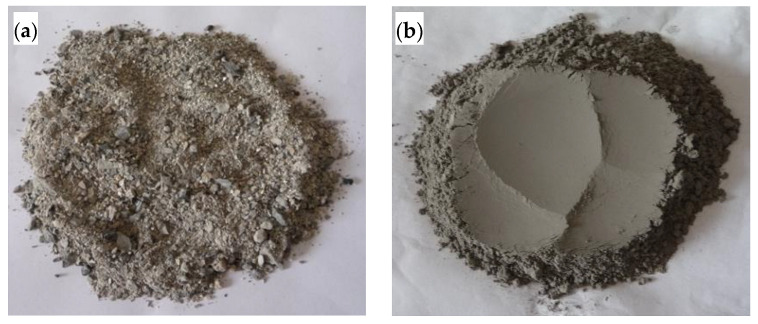
Digital images: (**a**) raw desulfurization slag; (**b**) ground desulfurization slag.

The raw desulfurization slag (RDS) was from Shanxi Pingshuo Coal Gangue Power Plant (Shuozhou City, Shanxi Province, China). The ground desulfurization slag (GDS) was prepared by grinding the RDS in a 5 kg mill for 39 min, which was used to prepare the SCCs in the experiment. The digital images of RDS and GDS are shown in Figure 1. The RDS is coarse and irregular. The specific surface area of the GDS is 400 m^2^/kg, its density is 2.61 g/cm^3^, its color is dark grey, and its main chemical composition is shown in Table 1. 

Grade P.O 42.5 Portland cement (PC) (Chihoi, Taiyuan City, China) was used to prepare the SCCs in the experiment. Its specific surface area is 370 m^2^/kg, its density is 3.1 g/cm^3^, and its main chemical composition is also shown in Table 1. The compressive strength of the cement mortar at 28d age is 50.3 MPa. Compared with PC, the GDS is lighter. 

The natural river sand (S) was used as fine aggregate, which was from Douluo Town (Xinzhou City, Shanxi Province, China). The apparent density is 2654 kg/m^3^, the bulk density is 1472 kg/m^3^, the mud content is 0.7%, and the fineness modulus is 2.8. The crushed limestone (CLS) was used as coarse aggregate, which was from Jiancaoping District (Taiyuan City, Shanxi Province, China). The particle size range is 5–20 mm, the mud content is 0.3%, the apparent density is 2786 kg/m^3^, and the bulk density is 1510 kg/m^3^. The polycarboxylate superplasticizer (PS) (Taiyuan concrete superplasticizer factory, Taiyuan, Shanxi Province, China) was used to improve the fluidity and the strength of the SCC, the water reduction rate of which was 34%. Sodium sulfate was used as activator to improve the GDS’s performance. The circular steel tubes with a straight slit were used to fabricate CFSTs, which were from Tangshan Iron & Steel Group (Tangshan City, Hebei Province, China).

### 2.2. Preparation of SCCs

The mix design and preparation of the SCCs were carried out according to JGJ/T 283-2012 “Technical Specification for Application of Self-compacted Concrete”. The GDS replaced cement in equal quantity. The water–binder ratio (W/B) of 0.3, 0.34 and 0.38, and the GDS’s amount of 0, 10%, 30% and 50% were used in the mix design. After trial and adjustment, the amount of each material is shown in Table 2.

### 2.3. Preparation of CFST Specimens

The parameters of the CFST specimens are shown in Table 3. Nine groups of specimens were designed with different SCC and different wall thickness of steel tube. SCC1~SCC7 shown in Table 2 were used to prepare the CFST specimens. Three different wall thicknesses (t) of the steel tubes were also used, which are 2 mm, 3 mm and 4 mm, respectively. The outside diameter (D) was 100 mm, and the length (L) of each specimen was 300 mm. Both the outside surface and the inside surface of the CFST specimens were cleaned and polished before use. A square steel cover plate of 2 mm thickness was welded to one end of the CFST specimens before casting SCC mixtures. The CFST specimens were poured without vibrations. After 24 h, the other end was welded to the CFST specimen. 

### 2.4. Filling Ability Test of Fresh SCCs

According to JGJ/T 283-2012 “Technical Specification for Application of Self-compacted Concrete”, the self-compacting properties of SCC include filling ability, passing ability and segregation resistance. Filling ability is measured by slump flow or slump-flow time, which is the control index. Passing ability is measured by the difference of slump flow value and J-ring flow value. Segregation resistance is measured by segregation percent and vibration segregation percent of coarse aggregate [30].

In this paper, the SCCs were used to fill steel tube as core concretes, which belong to concrete without steel bar. According to JGJ/T 283-2012, the filling ability of the used concrete needs to be tested, while the passing ability and segregation resistance do not require a test. Slump flow was tested to evaluate the filling ability of fresh SCCs in the test [31]. By adjusting the amount of the PS, the level of all SCCs’ filling ability reached SF1, which met the requirement of JGJ/T 283-2012.

### 2.5. Compressive Strength Test of SCCs

The concrete test blocks of compressive strength were produced at the same time as the CFST specimens, the size of which is 100 mm × 100 mm × 100 mm. After casting, they were put into the standard curing room along with the molds for curing, the mold was removed mold 1 day later, then the test blocks were wrapped with plastic film and put into an air-conditioned room with 20 ± 2 °C and 50% relative humidity for curing. The blocks were tested at 3d, 7d and 28d ages, respectively.

The test method and result evaluation were performed according to Chinese Standard GB/T 50107-2010 “Standard for evaluation of concrete compressive strength”.

### 2.6. Axial Compressive Test of CFST

A total of 9 groups of specimens mentioned in 2.3 were subjected to axial compressive test after 28d curing age. Before the test, four pairs of strain gauges were glued on the middle of the steel tube’s surface, which were used to obtain the axial strain and circumferential strain values [32].

**Figure 2:**
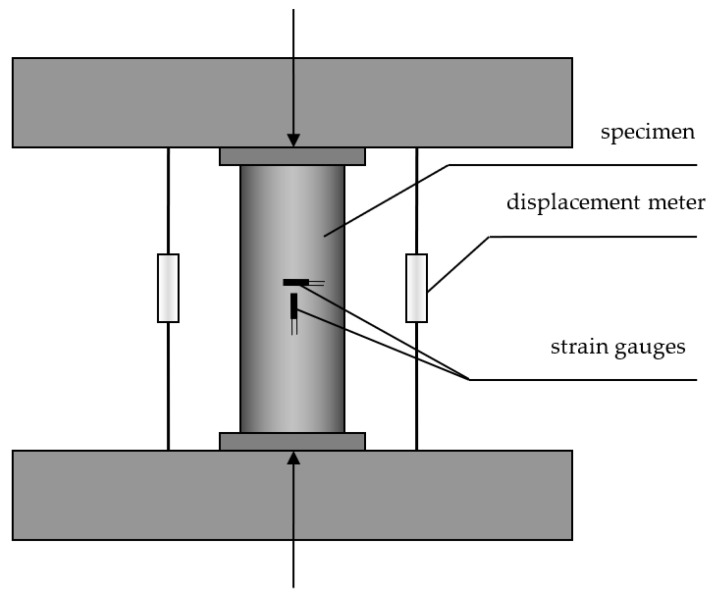
The test schematic diagram of CFST specimen.

The axial compressive test was carried out on the 3000 KN hydraulic test machine in Engineering Structure Laboratory of North University of China. The test schematic diagram is shown in Figure 2. The electrical displacement meters were used to test the axial deformation of the specimens. 

The load process of CFST specimens included two stages, which are the preload stage and the formal load stage. Before loading, the ultimate bearing capacity of each specimen was estimated according to CECS 28 “Technical specification for concrete-filled steel tubular structures”. In order to ensure the measuring instruments working normally, the preload was operated. In the preload stage, the load of 10% estimated ultimate bearing capacity was applied with a load rate of 1.0 kN·s^−1^. In the formal load stage, the load rate was 1.0 kN·s^−1^ initially. After loading to 80% of the estimated ultimate bearing capacity, the load rate reduced slowly to 1.0 kN·s^−1^ until the specimen was destroyed [33].

## 3. Results and Discussion

### 3.1. Filling Ability of the SCCs

**Figure 3:**
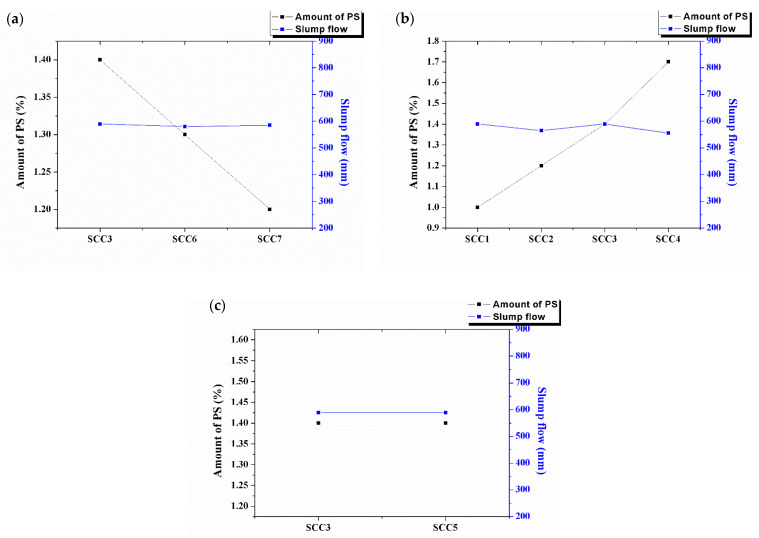
Different influence factors on amount of PS and slump flow of SCCs. (**a**) amount of GDS; (**b**) W/B; and (**c**) amount of sodium sulfate.

In order to facilitate the comparative analysis of concrete properties, the slump flow values were limited to a small range, which is 550 ~ 600 mm. Different influence factors on different amounts of PS and slump flow of SCCs are shown in Figure 3.

From Figure 3a, it can be seen that while the slump flow is almost constant, the amount of PS increases with the amount of GDS. The water demand of the desulfurization slag is larger than that of cement because of its coarse, loose and porous surface characteristics. Grinding can change its fineness but cannot change its surface characteristics. From Figure 3b, it can be seen that while the slump flow is almost constant, the amount of PS decreases with the water binder ratio. That is because when the water binder ratio increases, the water’s amount increases. In order to make the filling ability of the SCC constant, the amount of PS must be decreased. As shown in Figure 3c, the addition of sodium sulfate has little effect on the amount of PS and the slump flow of the SCC.

### 3.2. Compressive Strength of the SCCs

**Figure 4:**
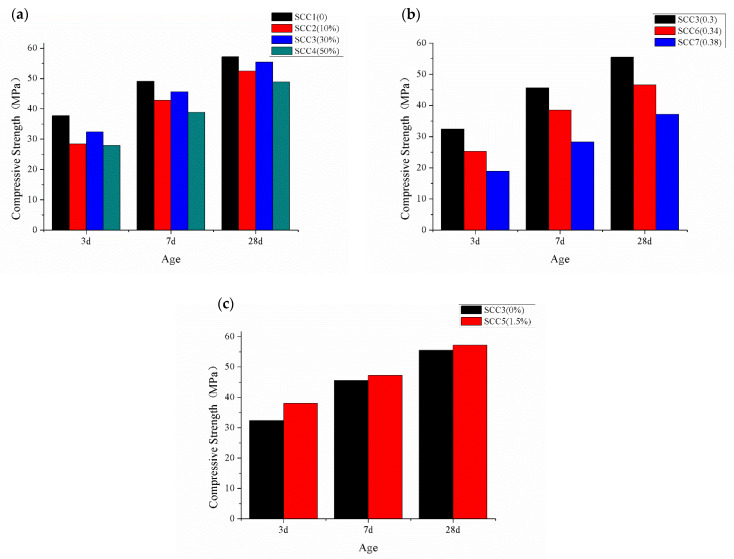
Different influence factors on the compressive strength at different ages. (**a**) amount of GDS; (**b**) W/B; and (**c**) amount of sodium sulfate.

Different influence factors on the compressive strength at different ages are shown in Figure 4.

From Figure 4a, it can be seen that with the increase in the GDS’s amount, the compressive strength decreases first, then increases and, finally, decreases at each age. All the compressive strength values of SCCs containing GDS are lower than that of SCC1 without GDS. The results indicate that the addition of GDS decreases the compressive strength of the SCCs. Compared with SCC1, the 28d compressive strength of SCC2, SCC3 and SCC4 decrease by 8.2%, 2.8% and 14.5%, respectively. The decrease rate of the 28d compressive strength of SCC3 is the minimum, which means that the optimum amount of GDS for SCCs is 30%. The desulfurization slag has much SiO_2_ and Al_2_O_3_. The GDS has good activity after grinding. There is also Ⅱ-CaSO_4_ in the GDS, which hydrates slowly and influences the increase rate of SCC’s strength [25]. From Figure 4b, it can be seen that with the increase in W/B, the compressive strength decreases at each age. The W/B is inversely proportional to the compressive strength, which is consistent with the water–cement ratio theory of ordinary concrete. The compressive strength of SCC6 and SCC7 are 16% and 33% lower than SCC3 at 28d age. As shown in Figure 4c, with the addition of 1.5% sodium sulfate, the compressive strength increases at each age. It means that addition of sodium sulfate can improve the SCC’s compressive strength. The 3d, 7d and 28d compressive strength increase by 17.6%, 3.5% and 2.9%, respectively. Relative to the late compressive strength, sodium sulfate improved the early compressive strength of SCC more pronounce. The addition of sodium sulfate can improve the early compressive strength of SCC, which is due to the activation of sodium sulfate on anhydrite existing in desulphurization slag. Sodium sulfate can speed the hydration of anhydrite and reaction with other substances [25]. 

### 3.3. Axial Compressive Properties of the CFSTs

**Figure 5:**
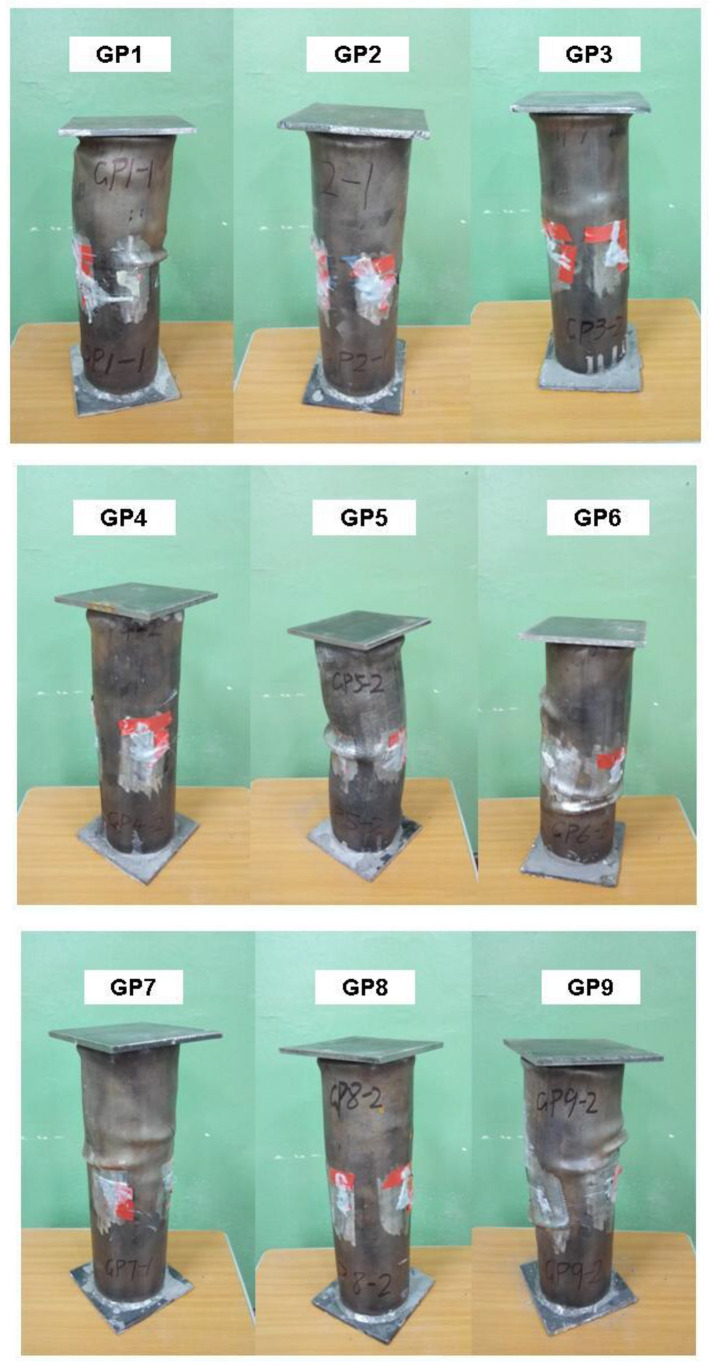
Typical failure form of specimens.

The typical failure form of specimens is shown in Figure 5. The failure form of the specimens is shear multi-fold waist drum failure, which is manifested as multiple waist drums formed along the axial direction on the surface of CFSTs. The failure mode of each specimen is ductility failure. At the initial loading stage, the CFST specimen is stable. When the loading continues, the upper and lower ends begin to buckle due to the edge effect. Then, shear slip appears and the middle gradually bulges. Continuing to load, the pressure gauge reading no longer rises until it is at the ultimate load [33]. 

**Figure 6:**
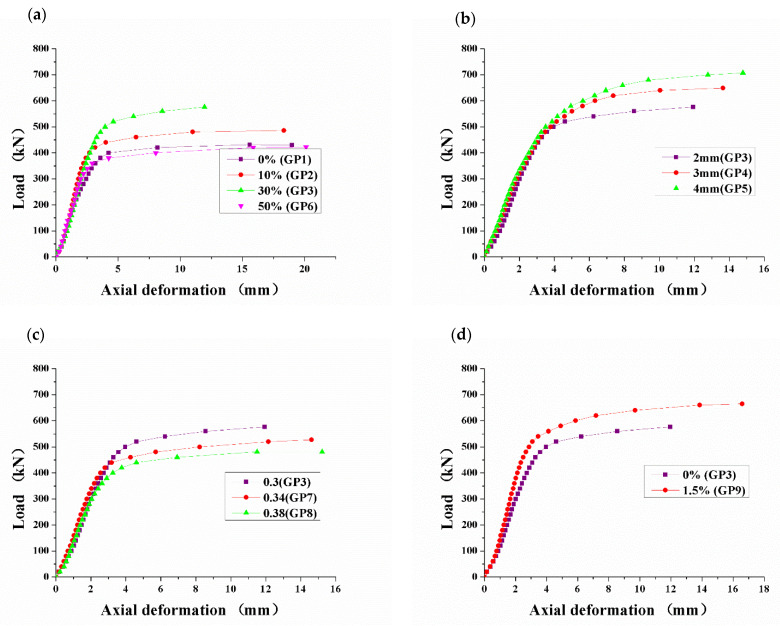
Different influence factors on the axial load–deformation of CFSTs. (**a**) amount of GDS; (**b**) wall thickness; (**c**) W/B; and (**d**) amount of sodium sulfate.

**Figure 7:**
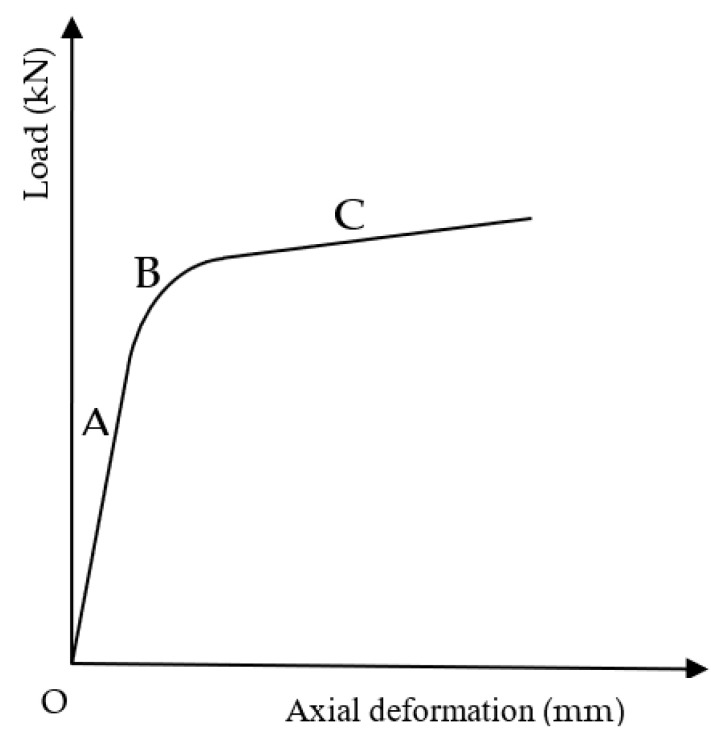
The axial load–deformation model curve of CFSTs. A—the elastic stage; B—the elastoplastic stage; C—the strengthening stage.

The axial load–deformation curves of CFSTs are shown in Figure 6. The axial load-deformation relation of all CFSTs conforms to the model curve in Figure 7, which can be divided into three stages [34]. The first stage (A) is the elastic stage, in which the relationship between load and deformation is close to linear, and the deformation is so small that it cannot be observed to the naked eye. The elastic ultimate load is about 80% of the total ultimate load. The second stage (B) is the elastoplastic stage, in which the load on the core concrete increases; while the load on the steel tube decreases, the deformation increase rate become faster than the load, and the load–deformation relationship deviates from the linear relationship, forming a nonlinear transition curve. The last stage (C) is the strengthening stage, in which the increased load is mainly borne by the core concrete. The lateral deformation of the core concrete increases rapidly, which pushes the steel tube in the radial direction. The tightening force of the steel tube on the core concrete produces, and the CFST’s bearing capacity is raised [33].

It can be seen from Figure 6a that the law of the axial load and the axial deformation of CFSTs with different amounts is basically consistent. The GP3 with a 30% amount of GDS has the largest elastic limit, the largest slope and the smallest deformation in the strengthening stage. The GP6 with a 50% amount of GDS has the lowest elastic limit, the lowest slope and the largest deformation in the strengthening stage. It can be seen from Figure 6b that despite the different wall thicknesses of CFSTs, the axial load–deformation curves have the same development law, with obvious elastic stage, elastoplastic stage and strengthening stage. The yield point of GP5 is the largest and the yield point of GP3 is the smallest, indicating that the larger the wall thickness is, the bigger the bearing capacity is. When the specimens fail, GP5 has the largest axial deformation, while GP3 has the smallest, mainly because that the steel tube with larger wall thickness has greater tightening force on the core concrete in the strengthening stage, which raises its bearing capacity. As shown in Figure 6c, W/B only affects the bearing capacity of CFSTs, and it has no influence on the load–deformation development law. When the specimens fail, GP8 has the largest axial deformation and the smallest bearing capacity, which is mainly caused by the low strength of the core concrete. It can be seen from Figure 6d that GP9 not only has a raised bearing capacity but also has a larger axial deformation when it fails, indicating that the ductility of CFST has been improved.

**Figure 8:**
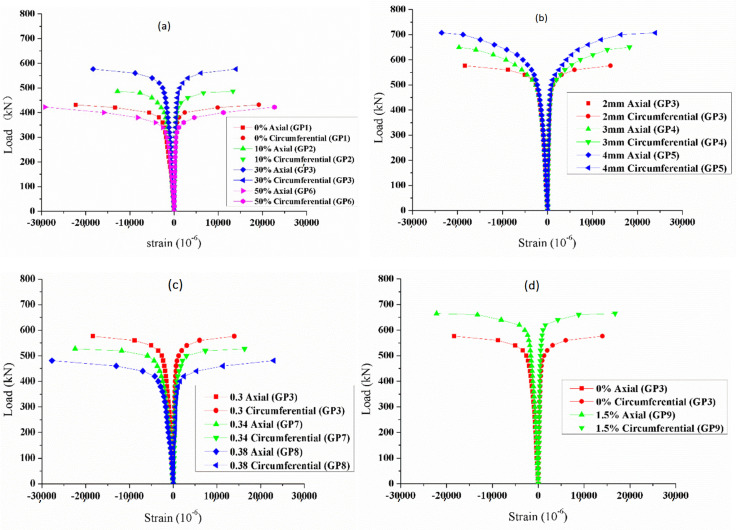
Different influence factors on the axial load–strain of CFSTs. (**a**) amount of GDS; (**b**) wall thickness; (**c**) W/B; and (**d**) amount of sodium sulfate.

The axial load–strain curves of CFSTs are shown in Figure 8. As shown in Figure 8, the load–strain relationship is basically consistent with the load–deformation relationship shown in Figure 6. The axial strain is greater than the circumferential strain when the specimens fail.

**Figure 9:**
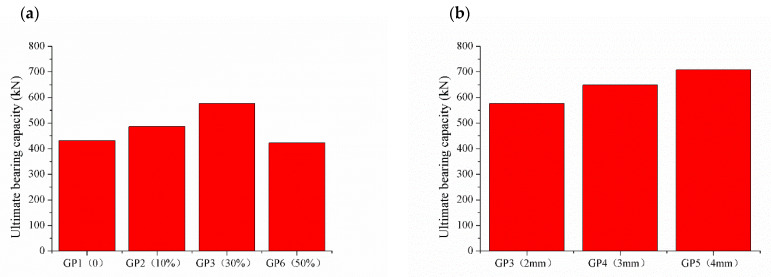

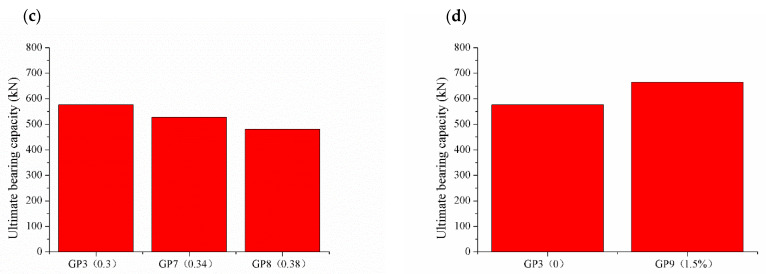
Different influence factors on the ultimate bearing capacity of CFSTs. (**a**) amount of GDS; (**b**) wall thickness; (**c**) W/B; and (**d**) amount of sodium sulfate.

The influence laws of different influence factors on the ultimate bearing capacity of CFSTs are shown in Figure 9. As shown in Figure 9a, GP1, GP2, GP3 and GP6 contains 0, 10%, 30% and 50% GDS, respectively. Although the amount of GDS increases, the ultimate bearing capacity of CFSTs first increases and then decreases. When the amount of GDS is 30%, the ultimate bearing capacity of CFST short column (GP3) is the highest, which is 33.6% higher than that of GP1 without GDS. Compared with GP1, the ultimate bearing capacity of GP2 with 10% GDS is 12.8% higher than that of GP1. The ultimate bearing capacity of GP2 with 50% GDS has the lowest ultimate bearing capacity, which is 2.1% lower than that of GP1. The optimum amount of GDS for CFSTs is 30%.

The influence law of the GDS’s amount on the CFSTs’ ultimate bearing capacity is quite different from that of the GDS’s amount on the SCCs’ compressive strength when comparing Figure 9a with Figure 4a. The ultimate bearing capacity of CFSTs can be significantly improved by adding GDS when the CFSTs are made with the same ratio as the SCCs. There are two reasons to explain why the CFSTs’ ultimate bearing capacity is raised. The first reason is that the desulphurization slag is coarse, irregular, loose and porous. Although it has been improved macroscopically after grinding, the microstructure characteristics have not been completely changed. Adding GDS into cement can improve the viscosity of the cementitious system [35]. When used to prepare SCCs, the cohesion of SCCs can be improved. Made into CFSTs, the good viscosity of the cementitious system can improve the interface binding force between the core concrete and the inner wall of the steel tube, so as to improve the ability of the steel tube and the core concrete to work together. The second reason is that the GDS has expansibility. The same with fly ash, desulfurization slag contains silicon oxide (SiO_2_) and aluminum oxide (Al_2_O_3_). The desulfurization slag also contains free calcium oxide (f-CaO) and anhydrite (II -CaSO_4_), which is different from the fly ash. The f-CaO reacts with H_2_O to form calcium hydroxide. The II-CaSO_4_ first dissolves to gypsum, then the gypsum reacts with calcium aluminate hydrate to form high sulfur calcium sulphoaluminate hydrate, commonly known as ettringite (AFt) [21]. Both of the two hydration reaction processes can lead to expansion. In the CFSTs, the GDS can compensate the shrinkage of the core concrete, even producing expansion to form the self-prestress of the core concrete when its amount is big. Under the restriction of the steel tube, the tightening force of the CFST is formed. The tightening force can improve the ability to resist deformation together between the steel tube and the core concrete at the initial loading stage, and prolong the elastic stage, thus improving the ultimate bearing capacity of CFSTs [36].

As shown in Figure 9b, with the increase in wall thickness, the ultimate bearing capacity increases. The ultimate bearing capacity of GP4 and GP5 are 12.6% and 22.7% higher than GP3. Increasing the wall thickness can increase the ultimate bearing capacity of CFST. The main reason is that in the elastic stage and the elastoplastic stage, the stress of CFST is mainly borne by the steel tube. The bigger the wall thickness, the greater the ability of resistance to deformation and the higher the elastic limit and yield point. 

As shown in Figure 9c, by comparison with GP3, GP7 and GP8, the ultimate bearing capacity of CFSTs decreases with the increase in W/B. That is because the increase in the W/B will decrease the compressive strength of the SCCs and then decrease the bearing capacity of the CFSTs. Compared with GP3, the ultimate bearing capacity of GP7 decreases by 8.5%, and the ultimate bearing capacity of GP8 decreases by 16.6%. Compared with the influence of W/B on compressive strength, the decline of the ultimate bearing capacity of CFSTs is significantly lower than that of the compressive strength of SCCs. The main reason is that the bigger the W/B is, the greater the self-stress of core concrete is and the greater the initial self-prestress exerted by the steel tube on it [37]. Due to the existence of initial self-prestress, the cooperative working capacity of the steel tube and the core concrete is improved, which compensates part of the bearing capacity decline caused by the increase in W/B.

As shown in Figure 9d, the ultimate bearing capacity of GP9 increases by 15.3%, compared with GP3. Compared with the influence of sodium sulfate on the compressive strength of SCC, which raised 2.9%, the bearing capacity of the CFST is greatly raised. The addition of sodium sulfate can increase the expansion of SCC, which is caused by hydration of anhydrite and the production of ettringite by chemical reaction after hydration. The SCC’s expansion raises the tightening force of the steel tube on the core concrete. As a consequence, the ability of the core concrete and the steel tube to work together is enhanced [33].

## 4. Conclusions

In this paper, the ground desulfurization slag is used to replace cement to prepare SCCs. Then, the SCCs are used as the core concrete of the CFSTs. Seven types of SCC are prepared and nine groups of CFST short column are fabricated. The filling ability and the compressive strength of the SCCs are researched, and the axial compressive properties of the CFSTs are analyzed. There are several conclusions that can be concluded:

- While the slump flow of the SCCs is almost constant, the PS’s amount increases with the GDS’s amount but decreases with W/B. The addition of sodium sulfate has little effect on the amount of PS and the slump flow of the SCC.
- The addition of GDS decreases the 3d, 7d and 28d compressive strength of the SCCs. The decrease rate of the 28d compressive strength of SCC3 is the minimum, at only 2.8%, which means that the optimum amount of GDS for SCCs is 30%. With the increase in W/B, the compressive strength of the SCCs decreases. The addition of sodium sulfate can improve the SCC’s compressive strength. The compressive strength of 3d, 7d and 28d increase by 17.6%, 3.5% and 2.9%, respectively. Relative to the late compressive strength, sodium sulfate improved the early compressive strength of SCC to be more pronounced.
- The failure mode of the CFST short column is ductility failure. The failure form of the specimens is shear multi-fold waist drum failure. The axial load–deformation relation curve of all CFSTs can be divided into three stages, which are the elastic stage, the elastoplastic stage and the strengthening stage.
- Although the amount of GDS increases, the ultimate bearing capacity of CFSTs first increases and then decreases. When the amount of GDS is 30%, the ultimate bearing capacity of CFST short column (GP3) is the highest, which is 33.6% higher than that of GP1 without GDS. When the amount of GDS is 10%, the ultimate bearing capacity of CFST short column (GP2) is 12.8% higher than that of GP1. When the amount of GDS is 50%, the ultimate bearing capacity of CFST short column (GP4) is 2.1% lower than that of GP1. The optimum amount of GDS for CFSTs is also 30%.
- The influence law of the GDS’s amount on the CFSTs’ ultimate bearing capacity is quite different from that of the GDS’s amount on the SCCs’ compressive strength. The ultimate bearing capacity of CFSTs can be significantly improved by adding GDS. With the increase in wall thickness, the ultimate bearing capacity also increases. The ultimate bearing capacity of CFSTs decreases with the increase in W/B. Compared with GP3, the ultimate bearing capacity of GP9 increases by 15.3%, which contains 1.5% sodium sulfate. Sodium sulfate can improve the bearing capacity of the CFST.

## Figures and Tables

**Table 1:** Main chemical composition of ground desulfurization slag and PC (%).

SiO_2_	Al_2_O_3_	CaO	SO_3_	Fe_2_O_3_	MgO	K_2_O	P_2_0_5_	Na_2_O	Loss
42.19	25.9	10.99	5.91	3.1	1.35	0.79	0.12	0.06	6.09
20.84	4.14	65.58	2.80	3.35	1.89	0.60	0.08	0.11	2.09

**Table 2:** The compositions’ amount of the SCCs (kg/m^3^).

No.	W/B	CLS	S	PC	GDS	Water	Sodium Sulfate	PS
SCC1	0.3	878	786	548	0.0	165	0	5.49
SCC2	0.3	878	786	493	55	165	0	6.58
SCC3	0.3	878	786	384	164	165	0	7.67
SCC4	0.3	878	786	274	274	165	0	9.32
SCC5	0.3	878	786	384	164	165	8.22	7.67
SCC6	0.34	878	786	366	157	178	0	6.79
SCC7	0.38	878	786	351	150	190	0	6.01

**Table 3:** Parameters of the CFST specimens.

No.	SCC	W/B	Amount of GDS (%)	Amount of Sodium Sulfate (%)	D × t × L (mm)
GP1	SCC1	0.3	0	0	100 × 2 × 300
GP2	SCC2	0.3	10	0	100 × 2 × 300
GP3	SCC3	0.3	30	0	100 × 2 × 300
GP4	SCC4	0.3	30	0	100 × 3 × 300
GP5	SCC5	0.3	30	0	100 × 4 × 300
GP6	SCC3	0.3	50	0	100 × 2 × 300
GP7	SCC3	0.34	30	0	100 × 2 × 300
GP8	SCC6	0.38	30	0	100 × 2 × 300
GP9	SCC7	0.3	30	1.5	100 × 2 × 300

## Data Availability

The data presented in this study are available on request from the corresponding author.
